# Surface albedo measurements and surface type classification from helicopter-based observations during MOSAiC

**DOI:** 10.1038/s41597-023-02492-6

**Published:** 2023-09-06

**Authors:** Tim R. Sperzel, Evelyn Jäkel, Falk Pätzold, Astrid Lampert, Hannah Niehaus, Gunnar Spreen, Sophie Rosenburg, Gerit Birnbaum, Niklas Neckel, Manfred Wendisch

**Affiliations:** 1 Leipzig University, Leipzig Institute for Meteorology, Leipzig, 04103 Germany; 2 TU Braunschweig, Institute of Flight Guidance, Brunswick, 38108 Germany; 3 University of Bremen, Institute of Environmental Physics, Bremen, 28359 Germany; 4 Alfred-Wegener-Insitute, Helmholtz Center for Polar and Marine Research, Bremerhaven, 27570 Germany

**Keywords:** Cryospheric science, Climate-change impacts

## Abstract

Global climate change poses significant societal and political challenges. The rapid increase in the near-surface air temperatures and the drastic retreat of the Arctic sea ice during summer are not well represented by climate models. The data sets introduced here intend to help improving the current understanding of the ongoing Arctic climate changes. In particular, this study considers observations from 24 helicopter flights (June–September 2020) and 5 flights with the helicopter-towed probe HELiPOD (May–July 2020) during MOSAiC. Distributions of various surface types (white ice/snow, bright melt ponds, dark melt ponds, open water, and bare ice) were determined using fisheye camera images. They were related to collocated broadband irradiance measurements to analyse the temporal and spatial changes of the surface albedo. Multiple linear regression was applied to assign the measured areal albedo to the corresponding surface-types. The resulting surface-type fractions, the albedo data and respective upward and downward broadband solar irradiances of several flights throughout the melting and refreezing season are provided.

## Background & Summary

The enhanced warming of the Arctic relative to the globe is commonly referred to as Arctic amplification^1,2^. Still, there are little-understood and under-investigated complex mechanisms driving Arctic amplification^3–6^. In the Arctic, long-term observational and *in situ* measurements are sparse despite their importance in the understanding of mechanisms and processes contributing to Arctic amplification^7,8^. During the Multidisciplinary drifting Observatory for the Study of Arctic Climate (MOSAiC), which took place from September 2019 to October 2020, these important data were collected^9–12^.

According to recent studies, the sea ice surface albedo feedback is a primary driver of Arctic amplification^13–15^. However, this feedback mechanism is not well represented in climate models, which significantly contributes to insufficiently accurate projections^16,17^. Typically, the surface albedo in climate models is parameterized based on snow and sea ice properties, such as skin surface temperature or snow depth^18,19^. However, the surface albedo parameterizations depend on further parameters, such as snow grain size, particle shape, and concentration of additional absorbing particles^20,21^. To include these parameters and, thus, improve existing albedo parameterizations, continuous measurements are necessary^22^.

**Fig. 1:**
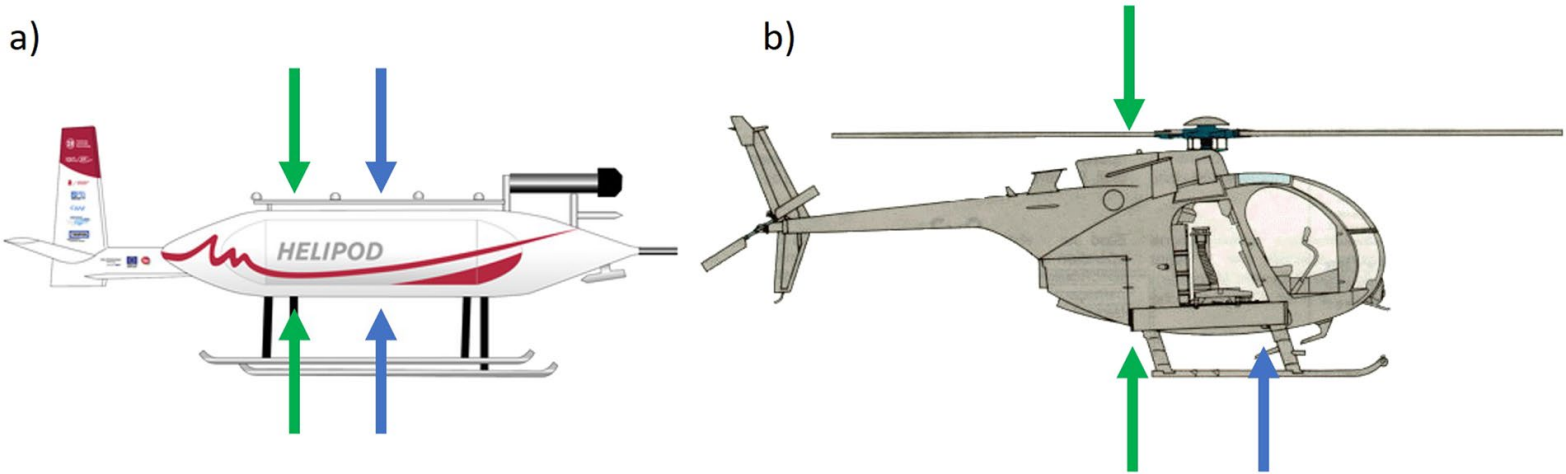
Measurement set-up for (**a**) HELiPOD and (**b**) helicopter. Green arrows indicate the location of CMP22 pyranometers, and blue arrows link to the position of Canon digital cameras. All instruments were installed facing in the direction of the arrow.

**Fig. 2:**
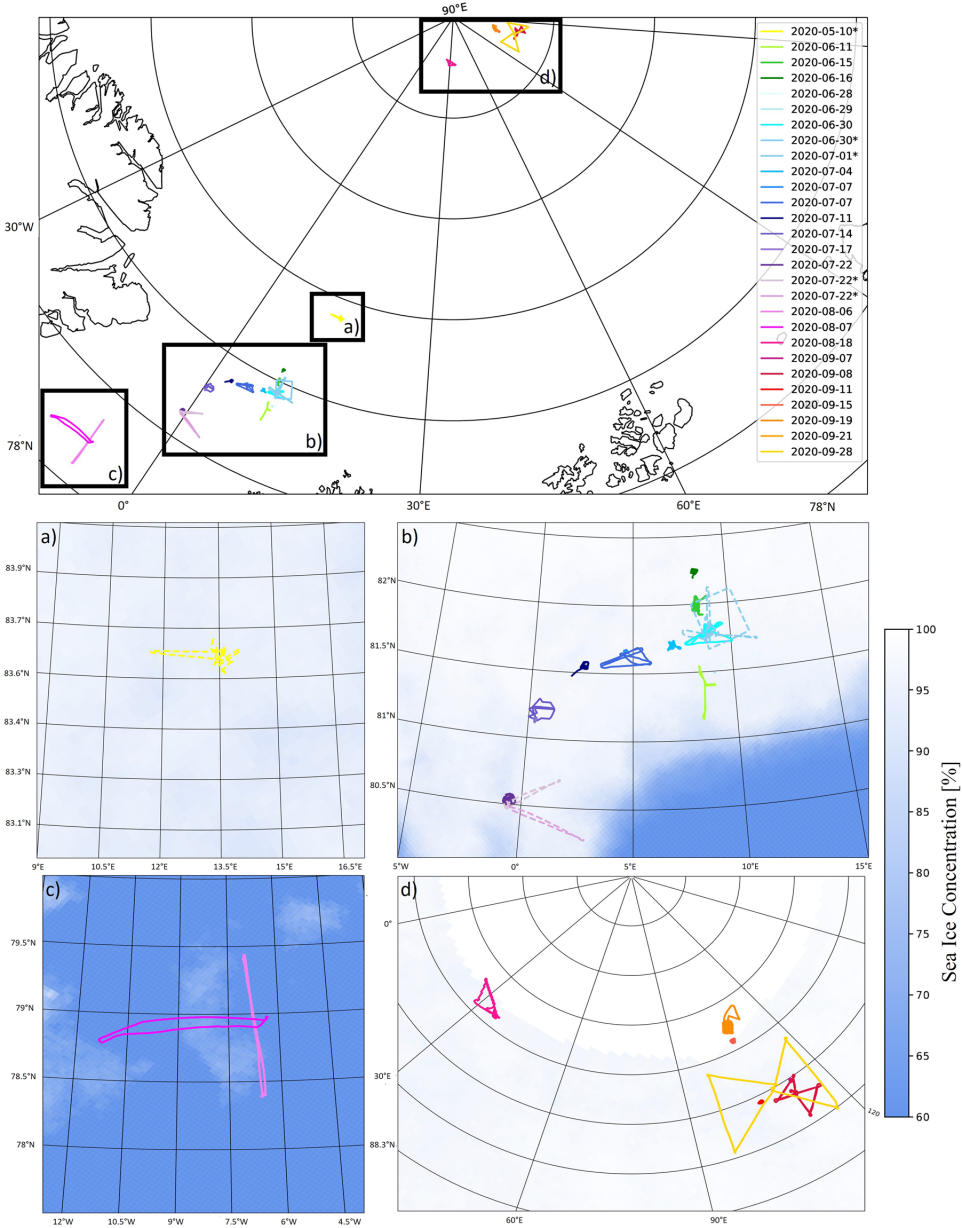
Flight path of all research flights (RFs) during MOSAiC. The panel on top provides a geographical overview; RFs conducted with HELiPOD are marked with an asterisk. The panels below introduce the RFs more detailed in respect to their respective sea ice concentrations (SICs). Panel (**a**) shows the SIC for May 2020; Panel (**b**) shows that for June and July 2020; Panel (**c**) shows that for August 2020; and Panel (**d**) shows that for September 2020. The HELiPOD flights in the four lower panels are indicated by the dashed lines.

During MOSAiC, the helicopter-borne probe HELiPOD conducted five research flights (RFs) from May to July 2020. The parameters used in this study comprise solar radiation components and images by a digital camera, which were used to identify surface types and distributions needed for further surface albedo-based analyses. The solar radiation components included broadband upward- and downward-facing irradiances using CMP22 pyranometers (manufacturer-calibrated) covering a spectral range from 210 nm to 3600 nm. For the digital images, a commercial Canon EOS RPs digital camera with fisheye lenses and a resolution of 6264–4180 pixels was used. Figure 1 indicates the location of all measurement devices relevant to this study. Green arrows show the position of the pyranometers; thus, blue arrows indicate the position of the cameras installed. Both devices were installed on the HELiPOD, as indicated in Fig. 1a and measured throughout the RFs simultaneously. Additionally, other essential data, such as the attitude of the HELiPOD, were collected. Images were taken every four seconds, and irradiances were measured at a frequency of 100 Hz. Besides HELiPOD, a second helicopter supported the measurements and conducted RFs as well. Figure 1b reveals that the helicopter was also equipped with two CMP22 broadband upward and downward-facing pyranometers (manufacturer-calibrated), measuring with 20 Hz frequency. However, the installed camera only faced downward, with a resolution of 3888–2592 pixels and an equipped fisheye lens^23^. The temporal resolution of the measurements from the helicopter was the same as for all measured parameters described for the HELiPOD. Note that during a HELiPOD RF was performed, no measurement instruments were installed at the helicopter but on the HELiPOD. The helicopter performed 24 RFs between June to September 2020. Figure 2 indicates the HELiPOD and helicopter flight paths and flight days with respect to the sea ice concentration (SIC)^24^. However, due to a lack of GPS data, the RF on June 14 is missing. In the top panel, all other 28 RFs are shown. The black boxes indicate sub-regions based on their location and the time of year. The lower four panels give a more detailed overview of the RFs within the boxes in relation to their average SIC. All flights took place above sea ice and started from the research vessel (RV) *Polarstern*^25^. An overview of all RFs and their data availability is given in Table 1. The HELiPOD RFs are marked by an asterisk. The average altitude is given, including their standard deviation *σ*. Pictures were taken with the fisheye cameras during all RFs. However, for several RFs, no radiation, GPS, or heading measurements are available yet; thus, these RFs can only be used for surface type classification. The atmospheric conditions were taken from the observations of the RV *Polarstern*^26^ and the images from HELiPOD. Note that the helicopter was not equipped with an upward-facing fisheye camera. The atmospheric conditions throughout the last 4 HELiPOD RFs were cloudless, however, the first RF of HELiPOD took place with a stable cloud cover. Clouds were alternating in between the helicopter RFs.

**Table 1:** All RFs considered.

Date	Time hh:mm:ss	Fisheye Camera	Radiation	Heading	GPS	Atmospheric Conditions	Avg. Altitude ±*σ*
2020-05-10*	11:20:06 - 11:45:54	X				Overcast	84.5 m ±54 m
2020-06-11	13:17:48 - 13:56:06	X			X	Overcast	163.6 m ±29 m
2020-06-14	09:21:46 - 10:50:46	X				Low level clouds	—
2020-06-15	12:10:32 - 13:30:04	X		X	X	Low level clouds	189.9 m ±16 m
2020-06-16	09:19:11 - 10:48:14	X		X	X	Overcast	194.9 m ±13 m
2020-06-28	08:51:20 - 09:24:04	X	X		X	Overcast	44.3 m ±15 m
2020-06-29	14:32:57 - 14:56:41	X	X		X	Overcast	51.7 m ±10 m
2020-06-30*	14:20:54 - 16:54:04	X				Cloudless	137,7 m ±290 m
2020-06-30	07:31:01 - 09:08:37	X	X	X	X	Cloudless	346.6 m ±19 m
2020-07-01*	11:43:26 - 15:10:02	X				Cloudless	151.3 m ±303 m
2020-07-04	08:42:50 - 10:33:14	X	X	X	X	Overcast	116.7 m ±20 m
2020-07-07	08:51:39 - 10:58:43	X	X	X	X	Overcast	99.7 m ±6 m
2020-07-07	12:25:52 - 14:27:20	X	X	X	X	Overcast	176 m ±15 m
2020-07-11	11:17:52 - 12:09:48	X	X	X	X	Overcast	199.8 m ±7 m
2020-07-14	11:57:50 - 13:27:38	X	X		X	Overcast	125.4 m ±21 m
2020-07-17	15:18:28 - 17:20:48	X	X	X	X	Overcast	100.7 m ±10 m
2020-07-22*	11:23:06 - 12:59:54	X	X	X	X	Cloudless	40.3 m ±31 m
2020-07-22*	12:59:58 - 15:35:52	X	X	X	X	Cloudless	27.9 m ±12 m
2020-07-22	15:12:28 - 17:31:56	X	X	X	X	Cloudless	335.9 m ±19 m
2020-08-06	08:51:40 - 10:56:14	X	X	X	X	Cloudless	227.3 m ±63 m
2020-08-07	08:33:02 - 10:22:23	X	X	X	X	Cloudless	314.3 m ±60 m
2020-08-18	11:41:42 - 16:25:43	X	X	X	X	Overcast	465.9 m ±1 m
2020-09-07	04:16:55 - 06:18:39	X	X	X	X	Cirrus	70.5 m ±1 m
2020-09-08	12:19:36 - 13:54:40	X	X	X	X	Overcast	464.3 m ±10 m
2020-09-11	05:27:22 - 07:42:50	X			X	Cloudless	573.8 m ±110 m
2020-09-15	04:35:26 - 06:22:22	X		X	X	Overcast	84.3 m ±12 m
2020-09-19	10:00:50 - 12:08:10	X		X	X	Overcast	298.6 m ±12 m
2020-09-21	07:41:36 - 09:45:12	X		X	X	Overcast	235.3 m ±64 m
2020-09-28	11:07:48 - 12:19:36	X		X	X	Overcast	142.3 m ±8 m

The dates marked by an asterisk indicate RFs conducted by HELiPOD. The X indicates availability of the measurement systems throughout the RFs. Average altitude is displayed with its standard deviation *σ*.

The introduced data sets consist of surface type fractions throughout all RFs and albedo and upward and downward irradiance measurements from 2 HELiPOD and 12 helicopter flights. They will be used in further studies, to evaluate existing surface albedo parameterizations and a new set of surface albedo parameterizations developed for specific climate models to improve future climate projections. Furthermore, the data sets can be used for additional cross-validations of other observational data gathered during the respective RF days.

## Methods

Processing the methodological steps described below produced two different data sets. The first concerns the classification of the different surface type fractions throughout the RFs of the HELiPOD and helicopter. The second contains the albedo values and radiation data. All methodological approaches were applied to both the HELiPOD and helicopter data sets.

### Surface type classification

The fisheye-lens cameras were calibrated with respect to their geometric characteristics using pictures of a square chessboard from different perspectives. These images were evaluated by an open source routine (http://opencv.org) implemented in computer vision algorithms to assign a viewing zenith and azimuth angle to each image pixel^27^. During data processing, the images were converted to a standardized format of 901–901 pixels, corresponding to a pixel resolution of about 0.2°. The visible parts of the HELiPOD skids or helicopter were marked in a black mask to exclude them from the data analysis. The RGB channels were separated for the first classification steps, and pixels with values < 5 in all three channels were marked as skids to be eliminated. A new mask for every RF was created to account for any modifications of the field of view between the RFs. With ten example pictures per flight serving as training samples, a visual examination was conducted to obtain the thresholds of RGB values to assign each image pixel to a specific surface type. Generally, white ice/snow, bright melt ponds, dark melt ponds, open water, and bare ice were distinguished. Areas within an image that clearly represent one of the surface types under the current atmospheric state were manually selected and analyzed with respect to the frequency distribution of the RGB values to define thresholds for the red, green, and blue channels. Since atmospheric and illumination conditions varied throughout all RFs, adapting the RGB thresholds of every single flight to the respective conditions was crucial. The total opening angle of the pictures regarding the surface type classification was restricted to 140°. The lens itself had an opening angle of 180°; however, it was discovered that the higher the opening angle, the higher the potential error in the RGB classification approach due to distortion. Furthermore, high absolute roll or pitch angles would cause misclassifications of the outer pixels. The total opening angle of 140° could capture a tremendous amount of surface area while avoiding wrongly classified pixels due to fisheye distortion. The classified surface type fractions were cosine-weighted by the viewing zenith angle (VZA) to account for the cosine-related response function of the pyranometers, as described by Wilks *et al*.^28^ (Eq. (1)):

1 $${f}_{{\rm{m}},{\rm{n}}}={\rm{\cos }}\left(\frac{{{\rm{VZA}}}_{{\rm{m}},{\rm{n}}}\cdot \pi }{180}\right),$$

where *f*_m, n_ represents the cosine-weighted fraction of the pixel (m,n) dependent on the VZA. This applied function ensured that parameterized fractions closer to the nadir point had a higher value than those with higher VZAs according to the pyranometers’ cosine response function.

**Fig. 3:**
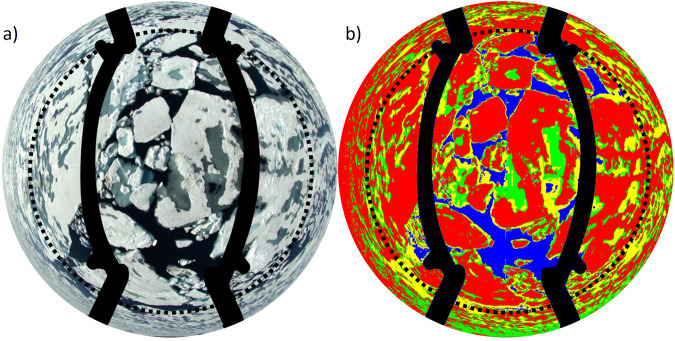
Visualized classification example of HELiPOD RF on July 22 2020). (**a**) A downward-facing picture example taken from HELiPOD with the typical fisheye distortion; (**b**), on the right, shows the corresponding classification. The red, green, yellow, and blue areas represent white ice/snow, bright melt ponds, dark melt ponds, and open water, respectively. Bare ice, however, is not present in this example. The masked skids are marked black and the dotted circle indicates the total opening angle of 140° considered for the classification.

Figure 3 illustrates this classification with a sample picture from a HELiPOD RF (2020-07-22). The original picture is displayed on the left, and the classified picture on the right. All surface type fractions are represented in the picture, except bare ice, which only occurs after the refreezing period starts. Unclassified pixels remain in the original pixel color and were considered in the classification values. The dotted circle indicates the total opening angle of 140°, considered the maximum for the classification. Classified pixels exceeding 140° opening angle were not considered due to distortion issues. Focusing on the surface outside the dotted circle would reveal obviously incorrect classifications upon comparing the pixels on the left with the classified ones on the right. Note that the results of the first HELiPOD RF (2020-05-11) should be taken with caution, as the green channel and part of the blue channel were overexposed throughout the flight. The overexposure was corrected by weighting the respective channels by 50% so that the surface structures became visible after correction.

### Derivation of surface albedo

Since the pyranometers were fixed to the helicopter/HELiPOD frame, it was crucial to correct the measurements for attitude changes. To account for the proportion of the direct incident radiation within the global radiation, libRadtran^29^ simulations were performed in 60 s temporal resolution using radiosondes of the respective RF day^30^. Following the description of Bannehr and Schiesow^31^, the proportion of the direct incident radiation was corrected using factor *k* (Eq. (2)):

2 $$k=\frac{\sin \left({h}_{0}\right)}{\cos \left({h}_{0}\right)\cdot \sin \left(\alpha \right)\cdot \sin \left({\phi }_{0}-h\right)-\cos \left({h}_{0}\right)\cdot \sin \left(\beta \right)\cdot \cos \left(\alpha \right)\cdot \cos \left({\phi }_{0}-h\right)+\sin \left({h}_{0}\right)\cdot \cos \left(\beta \right)\cdot \cos \left(\alpha \right)},$$

with solar altitude *h*_0_, and *α*, and *β* as roll and pitch offset angles, accordingly. However, only the direct incident radiation can be corrected, diffuse and upward (reflected) radiation can not be corrected. The incident direct radiation was weighted with *k* and combined with the proportion of diffuse incident radiation to obtain the final corrected downward irradiance (*F*_down_) (Eq. (3)):

3 $${F}_{{\rm{down}}}={F}_{{\rm{dir}}}\ast {f}_{u}\ast k+\left(1-{F}_{{\rm{dir}}}\right)\ast {f}_{u},$$

where *F*_dir_ represents the proportion of the direct incident radiation and *f*_u_ the measured, uncorrected global radiation. Both were scaled by factor *k*. By applying an attitude correction uncertainties and influences of the measurement system attitude throughout the RFs were considered for the direct component of the incident radiation. Note that the higher the absolute attitude angles, the higher the uncertainty of this method. Flights without available attitude data were excluded from the albedo retrieval. Furthermore, an offset in the roll and pitch measurements of the helicopter and HELiPOD was found and implemented in the attitude correction accordingly. According to Gröbner *et al*. the uncertainty of CMP22 pyranometers in stationary operation is 3%^32^. Due to a slow response of the CMP22 pyranometers a deconvolution was applied, following the descriptions of Ehrlich *et al*. and Ehrlich and Wendisch^33,34^.

The measured upward irradiance (*F*_up_) and attitude-corrected *F*_down_ data of the CMP22 pyranometers were combined with the respective RFs images. A lag correction of the corresponding time stamps was needed to connect the data sets. Therefore, the Pearson correlation was performed on the reflected radiation and the classified white ice/snow fraction. The results showed that no correction was needed for the helicopter data set; however, a lag of 21 s was found in the HELiPOD data set between the pictures and the CMP22 measurements. The lag was caused by two different time stamps used inside HELiPOD. One was made by the camera and could be calibrated every day. However, the second was created inside HELiPOD and assigned to the other measurements; therefore, only a calibration prior to the expedition was possible. The lag resulting from the two time stamps was corrected accordingly. After assigning a *F*_up_ and *F*_down_ value to each classified picture, the length of the combined data sets was restricted to the actual flight time, with no visual influence from the RV *Polarstern*. However, measurements during pictures of cloud covered RFs were assumed to be diffuse, and therefore assigned to uncorrected *F*_down_ values. Time steps with measured extreme *F*_down_ values exceeding the 99th percentile or falling below 1st percentile of the respective data set were sorted out due to unrealistically high/low values. Furthermore, as pictures including a sun glint could cause problems, especially regarding the surface type classification, all time steps with visible sun glint within the 140° opening angle were not considered further. To account for extreme attitude values and the previously described restricted possibilities to correct those corresponding *F*_down_ values, all time steps with an absolute roll or pitch value > 4.5° were eliminated for cloudless RFs. The threshold for non-cloudless RFs or flight paths was increased to 6.5°. Moreover, the *F*_down_ values were compared to simulations performed by libRadtran^29^ for all cloudless RFs, showing matching results. As no downward radiation data were available for the first three HELiPOD flights, these RFs were excluded from the albedo and radiation data set.

For the helicopter data set, further investigation revealed that the side rotor was causing issues in combination with the implemented CMP22 pyranometer when the relative azimuth angle to the sun was between 250° and 290°, within which a shading effect occurred influencing the *F*_down_ measurements during cloudless conditions. These measurements were eliminated or replaced by simulation values. Generally, the mentioned steps produce data gaps due to the described thresholds. In cloudless flight paths, these gaps were filled by simulations; data gaps for cloudy flight paths were ignored. All steps described also supported the elimination of measurements that did not fit the requirements for high-quality results. To provide the final albedo values for all RFs, the *F*_up_ and *F*_down_ values were taken as follows (Eq. (4)):

4 $${a}_{{\rm{t}},{\rm{p}}}=\left(\frac{{F}_{{\rm{up}},{\rm{p}}}}{{F}_{{\rm{down}},{\rm{p}}}}\right),$$

where *F*_up_ represents the reflected solar radiation measured by the downward-facing CMP22 pyranometer of time step *p* after the previously described steps and *a*_*t, p*_ illustrates the areal albedo^35^ per picture and time step *p*.

Finally, it was assumed that the areal albedo was a linear combination of *a*_*t, p*_ and surface type fraction *fr*_*p*_. Thus, multiple linear regression (MLR) was applied to the previously introduced albedo data and the classified surface type fractions to obtain the surface albedo per fraction and RF (Eq. (5)):

5 $${a}_{{\rm{t}},{\rm{p}}}=\mathop{{a}_{{\rm{ws}}}}\limits_{\_}\cdot {f}_{{\rm{ws}}}+\mathop{{a}_{{\rm{brmp}}}}\limits_{\_}\cdot {f}_{{\rm{brmp}}}+\mathop{{a}_{{\rm{dkmp}}}}\limits_{\_}\cdot {f}_{{\rm{dkmp}}}+\mathop{{a}_{{\rm{ow}}}}\limits_{\_}\cdot {f}_{{\rm{ow}}}+\mathop{{a}_{{\rm{bi}}}}\limits_{\_}\cdot {f}_{{\rm{bi}}},$$

where *f* indicates the cosine weighted proportion of the classified surface type fractions and *ws*, *brmp*, *dkmp*, *ow*, and *bi* represent the different surface type fractions of white ice/snow, bright melt ponds, dark melt ponds, open water, and bare ice, respectively. The underlined parameters are the regressed albedo values for the different surface type fractions of the corresponding RF.

## Data Records

All processed and described data sets have been published on PANGAEA (https://www.pangaea.de/). Raw data and scripts are stored on Zenodo https://zenodo.org/.

### Surface type classification

The surface type classification data^36^ are available as text files. For each flight, one text file is available (including daytime, longitude, latitude, altitude, solar zenith angle (SZA), roll, pitch, yaw, white ice/snow fraction, bright melt pond fraction, open water fraction, dark melt pond fraction, and bare ice fraction) for all pictures that went through the previously described quality checks. Time steps that not went through quality checks were eliminated. As shown in Table 1 not all parameters are available throughout all flights. Please note that those parameters are not included in the data set. Missing time steps are marked by “NA”. Please contact the corresponding author for access to unweighted surface type fractions. Images of RFs used for classification are publicly available in 901 × 901 format, as described^37^.

### Radiation and albedo

The radiation data^38^ are available as text files. The frequency for the radiation data set was sampled down to 10 Hz. In the final files for the radiation of every RF, further information is included considering the daytime, longitude, latitude, altitude, upward irradiance, and downward irradiance. Again, non-available parameters are not listed. Missing time steps are marked by “NA”. Time steps that not went through quality checks were eliminated. The albedo data set consists of the values displayed in Table 2, providing information about the albedo values per fraction after MLR for each RF. Radiation data before the quality check and MLR is publicly available^39^.

**Table 2:** The albedo values for all research flights (RFs) after multiple linear regression was applied.

Date	Time (UTC) hh:mm:ss	White Ice/Snow	Bright Melt Ponds	Dark Melt Ponds	Open Water	Bare Ice
2020-06-30	07:31:01 - 09:08:37	0.67	*0.32*	*0.07*	*0.02*	NA
2020-07-04	08:42:50 - 10:33:14	0.61	*0.32*	*0.07*	*0.02*	NA
2020-07-07	08:51:39 - 10:58:43	0.67	*0.31*	*0.07*	*0.02*	NA
2020-07-07	12:25:52 - 14:27:20	0.59	0.32	*0.07*	*0.02*	NA
2020-07-11	11:17:52 - 12:09:48	0.58	*0.32*	*0.07*	*0.02*	NA
2020-07-17	15:18:28 - 17:20:48	0.56	*0.32*	*0.07*	*0.02*	NA
2020-07-22*	11:23:06 - 12:59:54	0.58	0.24	0.07	0.02	NA
2020-07-22*	12:59:58 - 15:35:52	0.59	0.34	0.06	0.01	NA
2020-07-22	15:12:28 - 17:31:56	0.63	*0.32*	*0.07*	*0.02*	NA
2020-08-06	08:51:40 - 10:56:14	0.56	*0.32*	*0.07*	*0.02*	NA
2020-08-07	08:33:02 - 10:22:23	0.57	*0.32*	*0.07*	*0.02*	NA
2020-08-18	11:41:42 - 16:25:43	0.67	*0.32*	*0.07*	*0.02*	NA
2020-09-07	04:16:55 - 06:18:39	0.63	0.32	*0.07*	*0.02*	0.43
2020-09-08	12:19:36 - 13:54:40	0.69	*0.32*	*0.07*	*0.02*	*0.43*

The HELiPOD RFs are marked with an asterisk. The time refers to the flight time, and the albedo values per fraction represent the whole RF. Albedo values marked “NA” are nonexistent due to the not occurring of the corresponding fraction. Italic values represent fixed albedo values.

## Technical Validation

This section discusses the technical validation of the MLR performed. To validate the applied MLR method, three-dimensional (3-D) Monte Carlo simulations of radiative transfer were used to investigate a possible atmospheric 3-D effect. This effect is caused by horizontal photon transport due to multiple scattering by atmospheric gases, which may lead to a smoothing of the radiation field^40^. The MLR does not account for such 3-D effects when decoupling the areal surface albedo via linear combinations of the type-specific surface albedo. To identify possible 3-D radiative effects due to horizontal photon transport, radiative transfer simulations were running in two setups, one including atmospheric extinction, the other without. Moreover, standardized regression coefficients were utilized to examine the contribution of single variables to the MLR. Furthermore, an overview of the final derived data products is provided.

### Multiple linear regression

Radiative transfer simulations were performed via the Monte Carlo Atmospheric Radiative Transfer Simulator (MCARaTS), a 3-D Monte-Carlo-based radiative transfer model that can be used for simulating spectral irradiances^41,42^. As input for MCARaTS, equidistant grids (based on the pictures and classification) and spectral albedo values were needed. However, both were not provided by the measurement systems. For the pictures taken with the fisheye lens, the distortion needed to be removed, and for the radiation data sets, the broadband radiation needed to be transferred to spectral irradiance. Then, MCARaTS was applied to the three example pictures of the last HELiPOD RF on July 22 2020.

To remove the distortion, the previously described VZA in combination with the flight height during the RFs was used to compute the distance of each pixel to the nadir point within the opening angle of 140° (Eq. (6)):

6 $${d}_{{\rm{m}},{\rm{n}},{\rm{p}}}={h}_{{\rm{p}}}\cdot tan\left(\frac{\pi }{180\ast {{\rm{VZA}}}_{{\rm{m}},{\rm{n}}}}\right),$$

where d indicates the distance in meters to the nadir point of pixel m,n in picture p and *h*_p_ represents the flight height of the respective picture. The distances for every pixel in each picture were transformed into a coordinate system revealing the distance of each pixel on the x-axis and y-axis with respect to the nadir point and the viewing azimuth angle (VAA) (Eq. (7)):

7 $$\begin{array}{ccc}{x}_{{\rm{m}},{\rm{n}},{\rm{p}}} & = & {d}_{{\rm{m}},{\rm{n}},{\rm{p}}}\cdot {\rm{\sin }}\left(\frac{\pi }{180\ast VA{A}_{{\rm{m}},{\rm{n}}}}\right),\\ {y}_{{\rm{m}},{\rm{n}},{\rm{p}}} & = & {d}_{{\rm{m}},{\rm{n}},{\rm{p}}}\cdot {\rm{\cos }}\left(\frac{\pi }{180\ast VA{A}_{{\rm{m}},{\rm{n}}}}\right).\end{array}$$

**Fig. 4:**
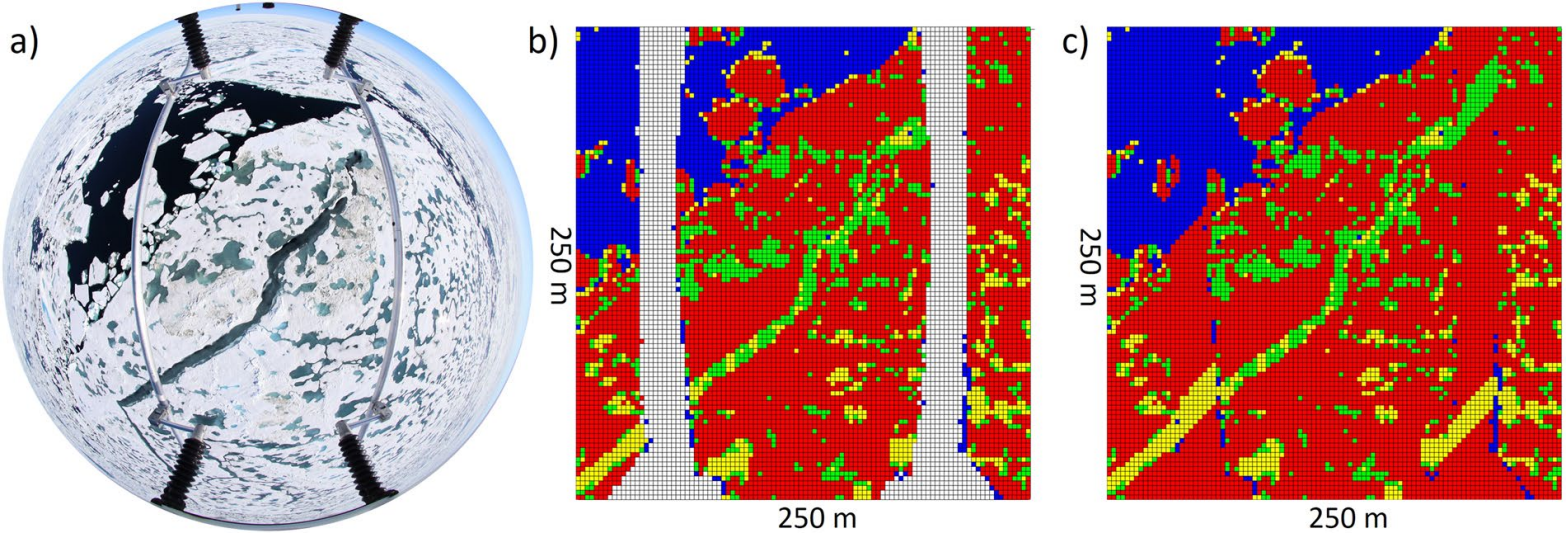
Examplary construction of equidistant grid cells based on fisheye distorted pictures. (**a**) on the left shows the original image; (**b**) indicates equidistant grids before nearest neighbor method; (**c**) indicates the final product used for the albedo input of MCARaTS, after applying nearest neighbor method. The colors indicate the mode of the different surface type fractions, replaced by the corresponding albedo values after MLR.

The mathematical signs were set according to the position of the pixel within the picture, assuming the nadir point as the center. Since the computing time of MCARaTS is affected by the resolution of the input, the resolution of the equidistant grids was lowered to a diameter of 250 m. Based on the coordinate system per pixel, a 100–100 matrix with a 2.5 m resolution was created, and the previously introduced albedo values depending on the classification, were assigned to their respective pixel. The closer the grid box was to the center, the fewer albedo values were considered for one grid, related to the fisheye distortion of the images. Since a mixing approach was not of interest, an interpolation could not be performed. Thus, the mode of all grid cells in the 100–100 matrix was selected to represent the respective cell. According to the previously applied mask and the selection of a 140° opening angle, only a few grids were not filled. The amount of unfilled grids strongly depended on the flight height during the RF since the coordinate system of the undistorted matrix was fixed. To fill the empty grids, a nearest neighbor method was applied. That is upon selection of the closest empty grid next to a filled grid, a virtual 10–10 box was opened, selecting all albedo values within and filling the empty grid by their mode. This step was repeated until all grids were filled for each RF image. Figure 4 visualizes the approach based on an example picture of the second HELiPOD RF on July 22 2020. On the left, an original image with fisheye distortion is displayed. By applying the introduced method to remove the distortion and create equidistant grid cells, a matrix, as displayed in the middle, was created (b). The color of the grids refers to the albedo of the surface type fractions obtained by classification, after MLR. Since the mask was applied before classification, the skids were masked out accordingly, resulting in empty grid cells. At c), on the right, the final result of the distortion removal after applying the nearest neighbor method is displayed. The previously empty grid cells were filled by the mode of the surrounding grids.

To estimate spectral albedo, the libRadtran simulations of the downward irradiance introduced previously for the HELiPOD RF on July 22 2020, were used since they revealed an agreement with the corrected radiation measurements. The libRadtran simulations were combined with the spectral albedo of different surface types (White ice/snow and melt ponds) from Istomina *et al*.^43^. Combining both data sets allowed estimating the upward irradiance for the HELiPOD RF from July 22 2020. Furthermore, the spectral surface albedo data set was scaled by a factor describing the relation of the broadband albedo per fraction after MLR and the integrated albedo per fraction of the introduced spectral albedo data set. Since MCARaTS simulates based on spectral data, the computing time might be long depending on the spatial, temporal, and spectral resolution. Thus, the band parameterization by Kato *et al*. was applied, dividing the solar spectrum into 32 spectral bands^44^. The spectral albedo was adapted to these bands accordingly.

**Fig. 5:**
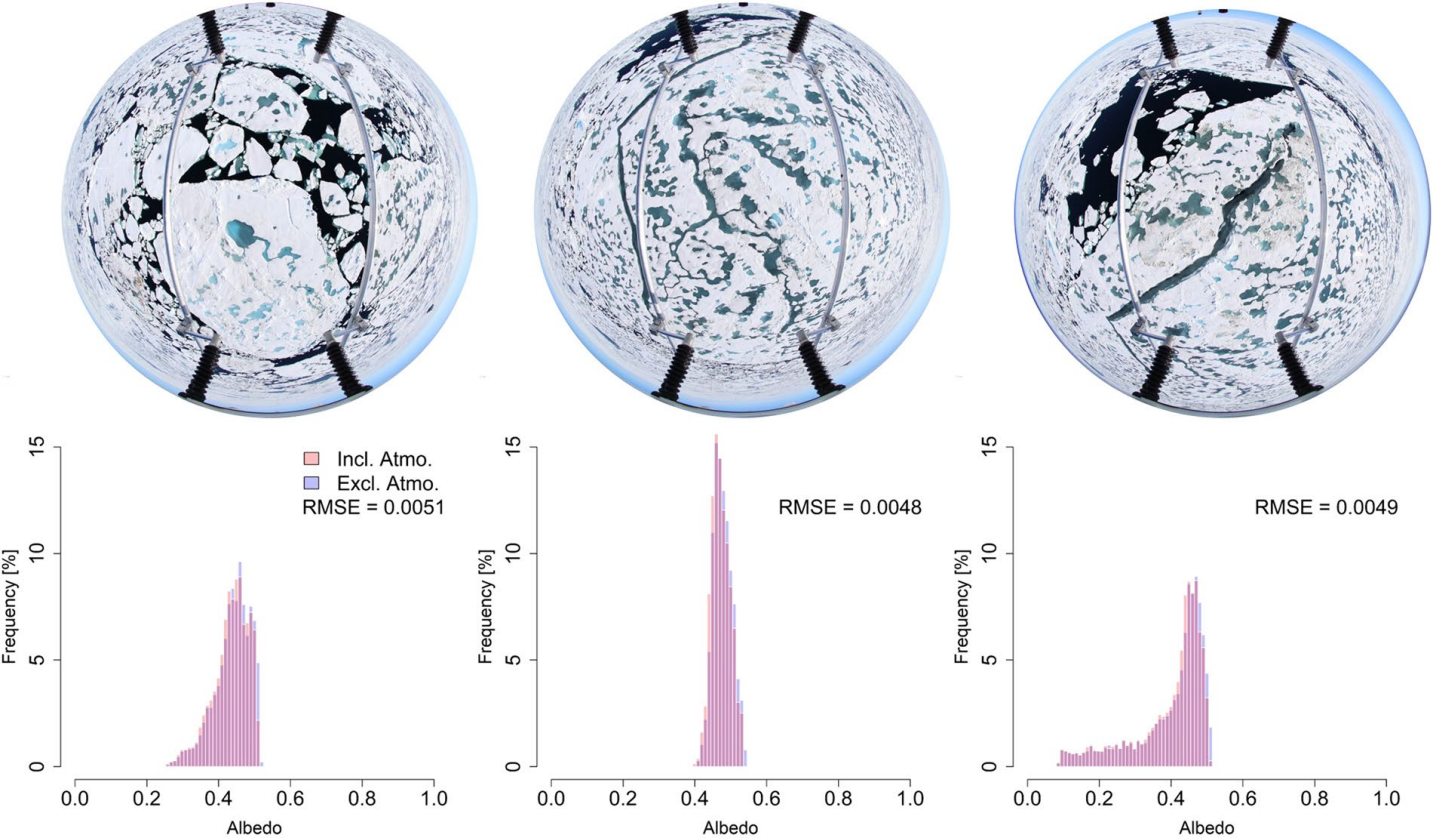
Results of MCARaTS. The top row shows the three pictures on which the radiative transfer simulations were based. At the bottom, the three respective albedo distributions are shown, including (red) or excluding (blue) atmospheric extinction.

Figure 5 displays a comparison of the two different MCARaTS runs. The top panel shows the corresponding RGB images, while the histograms at the bottom indicate the frequency of the simulated albedo values. By displaying the results with at least 100 bins, a slight shift of outer values more to the mean of the distribution is visible when adding atmospheric extinction. This slight shift can be accounted to the 3-D effects that affect MLR. However, analysis the respective distributions’ root-mean-square error (RMSE) revealed low values of between 0.0048 and 0.0051 for all pictures. Considering these results, it is suggested to neglect uncertainties related to 3-D radiative effects. Note that this applies only to the average flight height throughout the RFs used to prepare this data set.

After MLR was applied, standardized regression coefficients were calculated^45^ and evaluated. Only regressed values with a standardized coefficient > 0.5 were considered to account only for the main driving parameters of the MLR. Regressed albedo values that did not reach the threshold were fixed by the average albedo of the respective fraction of the HELiPOD RFs. The results showed that the contribution to the MLR was either very high and considered later or very low and replaced by a fixed value. Therefore, the fixed albedo values did not have a significant influence on the other considered and regressed albedo values.

To account for the uncertainty estimates of the MLR, a sensitivity study was applied by varying the surface type fractions ±10% and regressing again. Using the two fractions with the highest gradient regarding albedo (white ice/snow and open water) indicate the highest variability and, therefore, the highest uncertainty. Results showed that, on average, through all RFs the uncertainty accounts for about 1%.

### Derived products

**Fig. 6:**
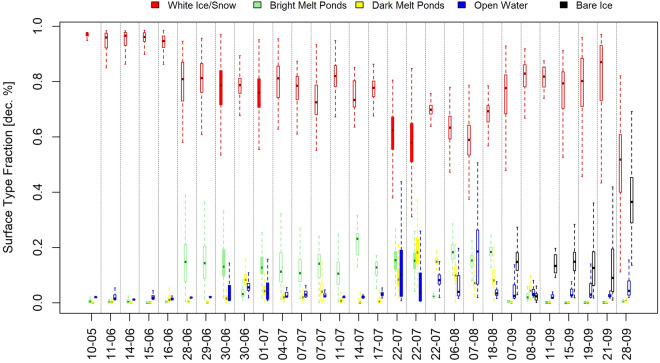
Surface type classification throughout all research flights (RFs) of the HELiPOD and helicopter. Filled boxplots indicate the HELiPOD surface type fractions, and unfilled boxplots indicate helicopter surface type fractions. The dotted lines separate the RFs accordingly.

Classification of the surface types data set was challenging due to the varying atmospheric conditions during the RFs. The final product was obtained by adapting the RGB thresholds used to the conditions of each RF. Figure 6 provides a summary of the cosine-weighted surface type fraction data set. All RFs are listed on the x-axis, while the corresponding fractions are displayed on the y-axis. Unfilled boxplots refer to helicopter RFs, whereas filled boxplots refer to HELiPOD RFs. The results were based on the images that passed all quality checks and were considered further for both data sets. The course of the surface type fractions throughout the RFs followed the expected distribution of the respective fractions. Snow/white ice decreased the further the melting season proceeded, and the melt pond and open water fractions increased accordingly. Note, that between the RFs on August 7, 2020, and August 18, 2020, the RV *Polarstern* moved further in the direction of the pole (see Fig. 1) as leg 5 of MOSAiC started. During the last RFs within leg 5, a shifted trend was visible, indicating that the refreezing period had started. The occurrence of another surface type, bare ice, supported this assumption, as refrozen melt ponds and leads are usually classified as bare ice.

**Fig. 7:**
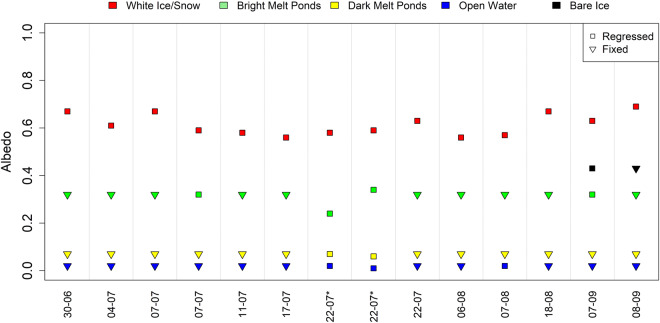
The albedo per RF divided into the classified fractions. Colors mark the corresponding surface type fraction as in the legend provided. Squares show regressed values, and triangles show fixed values.

As mentioned earlier, the radiation data set was limited to the available data and included fewer flights. In Fig. 7, a summary of the created data set is displayed. All available RFs are listed on the x-axis in temporal order, indicating the albedo per fraction as a summary of the RF itself. During all RFs, the albedos of the different regressed fractions seemed stable, atypical for the melting processes during spring and summer. Note that the temporal comparison between the RFs themselves should be taken with caution due to the inconsistent atmospheric conditions and the drift of the RV *Polarstern* throughout the RFs. This inconsistency explains why the typical negative trend throughout the melting process was not represented within the data set. Squares mark albedo values that were regressed, as discussed previously. Triangles, however, indicate fixed albedo values based on the mean respective surface type albedo of all HELiPOD flights. Albedo values were fixed if the distribution of the data was misleading for the MLR approach or the previously mentioned standardized regression coefficient was below the threshold. Only bare ice was fixed based on the RF of September 7 since it did not occur during the HELiPOD RFs.

## Usage Notes

All data sets introduced in this manuscript are publicly available for download and use. As the authors are continuously working with the introduced data sets, it might be useful to point out planned research and methodologies on the data sets to share known issues that come up after the publication of the manuscript. All introduced data sets are available in common data formats. Only common programming software is needed to open and handle the data. In case of further questions, please contact the corresponding author via E-Mail.

### Surface type classification

Note that due to the previously described sortings and quality checks, not all pictures taken during the RFs were implemented in the final product. Those pictures not matching the quality requirements, such as the attitude of the measurement device during flight, were excluded. This has caused temporal gaps in the final product, which can be identified by the column of the data set indicating the daytime. All classified values correspond to a total opening angle of 140°. For the helicopter RFs, the 140° opening angle covers the total visible surface without any cutoff since parts of the installed camera were surrounded by parts of the helicopter. Note that the images are not included in the data set due to saving capacities. Please contact the corresponding author for access to the original or visually classified pictures.

### Albedo and radiation

The albedo data set does not include as many RFs as the surface type classification data set because of the data availability issues mentioned previously. The albedo values were only determined for pictures and measurements that passed the quality checks. Only measurements outside of the optical effect of the RV *Polarstern* or other equipment were considered. Note that the albedo values per fraction after MLR were based on the described surface classification with a 140° opening angle.

For the radiation data set, the offset angles of all RFs in both measurement systems were adapted for every flight manually since it could not be ensured that the measurement devices were not removed and reinstalled after and before the respective flight days. Thus, for the attitude correction, the offset was not always the same. For further information or detailed offset values, please contact the corresponding author. If requested, the authors can provide the radiation in higher/lower frequency.

### Application examples

The introduced surface type classification or albedo and radiation data sets could be further used for evaluation purposes. Many climate models still struggle to realistically represent the Arctic climate and sea-ice fractions and corresponding albedo values. In climate projections simulations, these uncertainties can produce unreliable results and systematically underestimate the Arctic amplification and the concomitant effects^46^. The introduced data sets will help validate existing albedo parameterization schemes within climate models. Those climate models that not only parameterize the albedo within the scheme but also the surface type fraction, e.g., HIRHAM-NAOSIM, will even benefit from the surface type classification when it is about validation^17^. The surface type classification can also be used to validate satellite data in remote sensing. Typically retrieving surface classification from satellite data is difficult due to its lower spatial resolution with a limited possibility to capture small-scale differences based on the methods used. A recent study introduced Arctic-wide sea ice melt pond fractions derived by Sentinel-2 data^47^. Comparing and validating this or similar data sets based on remote sensing methods with the introduced data sets in this manuscript would divulge interesting results and might help to further improve existing classification methods using datasets of lower spatial resolution.

## Data Availability

The surface classification and attitude correction of the radiation data set was performed in the IDL software (version 8.8.3). Multiple linear regression and quality checks in addition to the MCARaTS simulations were coded in R (version 4.0.4) using the libraries: zoo (version 1.8.9) and gridExtra (version 2.3). The MCARaTS simulations were prepared using R (version 4.0.4) in combination with the libraries readr (version 1.4), jpeg (version 0.1.9), png (version 0.1.7), and stringr (version 1.4). The simulations were performed by MCARaTS (version 0.1). Code for classification of the surface type fractions and connection, quality check and MLR of the radiation data sets are publicly available^48^.
